# Epigenome-Wide DNA Methylation in Unipolar Depression: Predictive Biomarker of Antidepressant Treatment Response?

**DOI:** 10.1093/ijnp/pyae045

**Published:** 2024-10-05

**Authors:** Miriam A Schiele, Oscar Crespo Salvador, Jan Lipovsek, Kathrin Schwarte, Pascal Schlosser, Peter Zwanzger, Volker Arolt, Bernhard T Baune, Anna Köttgen, Katharina Domschke

**Affiliations:** Department of Psychiatry and Psychotherapy, Medical Center – University of Freiburg, Faculty of Medicine, University of Freiburg, Freiburg, Germany; Department of Psychiatry and Psychotherapy, Medical Center – University of Freiburg, Faculty of Medicine, University of Freiburg, Freiburg, Germany; Department of Psychiatry and Psychotherapy, Medical Center – University of Freiburg, Faculty of Medicine, University of Freiburg, Freiburg, Germany; Institute of Genetic Epidemiology, Faculty of Medicine and Medical Center – University of Freiburg, Freiburg, Germany; Department of Psychiatry and Psychotherapy, University of Münster, Münster, Germany; Centre for Integrative Biological Signalling Studies (CIBSS), University of Freiburg, Freiburg, Germany; Department of Epidemiology, Johns Hopkins University Bloomberg School of Public Health, Baltimore, Maryland, USA; Institute of Genetic Epidemiology, Faculty of Medicine and Medical Center – University of Freiburg, Freiburg, Germany; Department of Psychiatry and Psychotherapy, Ludwig-Maximilians-University of Munich, Munich, Germany; kbo-Inn-Salzach-Klinikum, Wasserburg am Inn, Germany; Institute for Translational Psychiatry, University of Muenster, Muenster, Germany; The Florey Institute of Neuroscience and Mental Health, The University of Melbourne, Parkville, VIC, Australia; Department of Psychiatry, Melbourne Medical School, The University of Melbourne, Melbourne, Australia; Department of Psychiatry and Psychotherapy, University of Münster, Münster, Germany; Institute of Genetic Epidemiology, Faculty of Medicine and Medical Center – University of Freiburg, Freiburg, Germany; German Center for Mental Health (DZPG), Partner Site Berlin, Berlin, Germany; Department of Psychiatry and Psychotherapy, Medical Center – University of Freiburg, Faculty of Medicine, University of Freiburg, Freiburg, Germany

**Keywords:** EWAS, methylome, epigenetic, MDD, depression, SSRI

## Abstract

**Background:**

Despite the well-documented efficacy of antidepressant agents for the treatment of major depressive disorder (MDD), initial treatment nonresponse rates are high. Recent years have seen an increase in research into predictive biomarkers toward improving diagnosis and individualized treatment. Among those, epigenetic mechanisms such as DNA methylation constitute promising candidate markers in predicting antidepressant treatment response in MDD. The present study sought to address epigenome-wide DNA methylation as a predictor of antidepressant treatment response in the largest sample to date of patients with MDD.

**Methods:**

Epigenome-wide DNA methylation was analyzed using the Infinium MethylationEPIC BeadChip in peripheral blood of n = 230 Caucasian patients with MDD receiving 6-week antidepressant treatment in a naturalistic in-patient setting as well as in a subsample of n = 107 patients primarily receiving continuous treatment with serotonin reuptake inhibitors or serotonin and norepinephrine reuptake inhibitors. Treatment response was assessed by means of the Hamilton Depression Scale.

**Results:**

No genome-wide significant hits were observed. Suggestive (*P* < 1E-5) epigenome-wide evidence was discerned for altered DNA methylation at 6 CpG sites (*LOC102724467*, *LOC100506023*, *RSPO2*, *SAG*, *IL16*, *PRKCI*) to predict response to naturalistic antidepressant treatment. In patients treated with serotonin reuptake inhibitors or serotonin and norepinephrine reuptake inhibitors, differential DNA methylation at 11 CpGs, for example, mapping to the *TIMP2*, *VDAC1*, or *SORL1* genes, was suggestively associated with treatment response.

**Conclusions:**

The present results provide preliminary evidence for altered DNA methylation patterns to be associated with antidepressant treatment response in MDD. Provided significant replication in independent and larger samples, the present findings might in the future aid in clinical decision-making toward more individualized and thus more efficacious treatments of MDD.

**Significance Statement**

Treatment nonresponse poses a significant challenge to effectively managing major depressive disorder (MDD) and highlights the need for identifying predictive biomarkers to guide personalized treatment approaches. Recent research has increasingly focused on epigenetic mechanisms, particularly DNA methylation, to predict antidepressant response. The present study aimed to explore the predictive value of epigenome-wide DNA methylation profiling in a large cohort of patients with MDD undergoing antidepressant treatment. Suggestive evidence emerged for altered methylation patterns at 6 CpG sites to predict response to a naturalistic antidepressant treatment and at 11 CpGs in patients treated with SSRIs or SNRIs. Overall, the present study provides additional insights into the potential of DNA methylation to predict antidepressant treatment response in MDD, suggesting avenues for further investigation toward more individualized and potentially more efficacious treatment strategies.

## INTRODUCTION

Major depressive disorder (MDD) is associated with a significant burden of disease, accounting for 37% of disability-adjusted life years attributed to mental disorders (GBD Mental Disorders Collaborators,  2019), and high chronicity (Verduijn et al., 2017), which is partly also due to treatment resistance. Despite the well-documented efficacy and tolerability of serotonin reuptake inhibitors (SSRIs) and serotonin and norepinephrine reuptake inhibitors (SNRIs), initial treatment nonresponse rates in MDD are reported to be as high as 60% (Fava, 2003). This has spurred research into identifying early biomarkers for the prediction of antidepressant treatment response in an effort to provide personalized and thus more effective treatment options (Bredt et al., 2015; Mora et al., 2018; Abi-Dargham et al., 2023). Among candidate biomarkers, recent years have seen an increase in attention to the involvement of epigenetic processes in predicting and possibly also mediating treatment response in affective disorders (Menke et al., 2012; Vialou et al., 2013; Nestler et al., 2016; Schiele et al., 2020). Epigenetic processes such as DNA methylation crucially modulate gene function without, however, entailing changes to the DNA sequence itself (Moore et al., 2016; Schuebel et al., 2016). Previous epigenetic investigations of antidepressant treatment response have primarily followed candidate gene–based approaches and focused mainly on well-described candidate genes such as *BDNF*, *NR3C1*, and *FKBP5* or the serotonergic and noradrenergic system (e.g., Menke and Binder, 2014; Chen et al., 2017; Webb et al., 2020). Thus far, only 4 pilot studies have investigated genome-wide DNA methylation patterns for their potential as predictors of response to pharmacological antidepressant treatment: Takeuchi et al. (2017) identified significantly altered methylation at 2 CpGs in the genes coding for Liprin-Alpha-4 (*PPFIA4*) and Sulfate-Glucosamine 3-Sulfotransferase 1 (*HS3ST1*) to be related to response to 6-week paroxetine treatment in 10 responders compared with 10 nonresponders in a sample of Japanese patients with MDD. Another study observed 3 differentially methylated positions upstream of the Chimerin 2 (*CHN2*) and Janus Kinase 2 (*JAK2*) genes between n = 82 responders and n = 95 nonresponders to 8-week escitalopram treatment in MDD patients (Ju et al., 2019). In n = 22 depressed children and adolescents receiving fluoxetine treatment for 8 weeks, differential methylation at 21 CpG sites, for example, at the Ras Homolog Family Member J (*RHOJ*) and Olfactory Receptor family 2 subfamily L member 13 (*OR2L13*) gene loci (Martinez-Pinteno et al., 2021), was discerned. Finally, Engelmann et al. (2022) observed no genome-wide significant but a nominal association of 20 differentially methylated regions in or near genes coding for, for example, the Sorbin And SH3 Domain Containing 2 (*SORBS2*) and Cytochrome P450 Family 2 Subfamily C Member 18 (*CYP2C18*) genes with treatment response to SSRIs/SNRIs in n = 40 treatment responders compared with n = 40 nonresponders with MDD.

The present study aimed at identifying DNA methylation patterns associated with differential antidepressant treatment response in a hypothesis-generating, genome-wide approach in the largest sample to date of MDD patients treated in a naturalistic setting as well as in a subsample treated primarily and consecutively with SSRIs or SNRIs.

## METHODS

### Sample

A total of n = 236 patients with MDD (138 female; mean age ± SD: 48.26 ± 15.90 years; Schiele et al., 2021) were recruited at the Department of Psychiatry and Psychotherapy, University of Muenster, Germany, between 2004 and 2011 (cf. Schiele et al., 2021). Patients received antidepressant treatment in a naturalistic inpatient setting. Study inclusion criteria were diagnosis of MDD according to DSM-IV criteria (assessed via SCID-I interview), antidepressant treatment for at least 6 consecutive weeks, and European ancestry. Patients receiving monoamine oxidase inhibitors, valproate, or concomitant electroconvulsive therapy were excluded. Diagnosis of bipolar disorder, cyclothymia, psychotic disorders including schizoaffective disorder, comorbid substance abuse or addiction, intellectual disability, or severe somatic or neurological disorders led to study exclusion (cf. Domschke et al., 2014, 2015; Schiele et al., 2021). Symptoms of depression were assessed weekly by means of the Hamilton Depression Scale (HAM-D 21). Ethical approval was obtained from the ethical board of the University of Muenster, Germany. Written informed consent was obtained from all patients, and the study was conducted in agreement with the ethical principles of the Declaration of Helsinki in its latest version.

### Medication

All patients received antidepressant treatment in a naturalistic study design (cf. Schiele et al., 2021). In detail, in the overall sample as assessed in treatment week 1, n = 69 patients were treated with SNRIs, n = 32 with SSRIs, n = 32 with tri- or tetracyclic antidepressants, n = 85 with a noradrenergic and specific serotonergic antidepressant (partly in subclinical dosage for enhancing sleep), n = 8 with a norepinephrine reuptake inhibitor (NRI), and n = 1 with a norepinephrine-dopamine reuptake inhibitor. Medication was given as either stand-alone or combination treatment. Co-medication with atypical (n = 77) and typical antipsychotics (n = 12), anticonvulsants (n = 13), lithium (n = 15), benzodiazepines (n = 61), or zopiclone/zolpidem (n = 17) was used as partly off-label augmentation of the primary antidepressant treatment (cf. Schiele et al., 2021).

Of the overall sample, n = 110 patients received either an SSRI or SNRI as their primary antidepressant continuously for 6 weeks (initiation of SSRI/SNRI treatment no later than the second week of in-patient treatment to account for the delay in onset of effect) and were considered for subgroup analysis (cf. Schiele et al., 2021). Of those, n = 14 received continuous treatment with an SSRI or SNRI only. Co-medication in the remaining sample comprised tetracyclic antidepressants (n = 14), noradrenergic and specific serotonergic antidepressant (n = 57; partly in subclinical dosage for sleep enhancement), NRI (n = 6), atypical (n = 70) and typical antipsychotics (n = 5), anticonvulsants (n = 11), lithium (n = 6), benzodiazepines (n = 36), or zopiclone/zolpidem (n = 13).

### Epigenome-wide DNA methylation data and statistical analysis

EDTA-blood was taken at admission. DNA was isolated using the FlexiGene DNA Kit (QIAGEN, Hilden, Germany) and stored at −80°C until further processing. After bisulfite conversion, DNA methylation at approximately 865 000 sites was quantified by means of the Infinium MethylationEPIC Kit (Illumina, San Diego, CA, USA). Bisulfite conversion, hybridization, and processing were performed according to the manufacturer’s instructions at Life & Brain, Bonn, Germany. Similar to previous studies (cf. Ziegler et al., 2019; Schiele et al., 2022), raw methylation data were processed and quality-controlled with a modified version of the CPACOR pipeline (Lehne et al., 2015). The proportions of WBC type composition (CD8 T cells, CD4 T cells, natural killer cells, B-cells, monocytes, granulocytes) were estimated by the Houseman method (Houseman et al., 2012; Fortin et al., 2017). After data preprocessing and quality control, DNA methylation data and complete clinical data were available for treatment prediction analyses from n = 230 naturalistically treated MDD patients and from a subsample of n = 107 MDD patients treated with SSRIs or SNRIs. DNA methylation β-values were used as predictors of interest, and change in depression symptoms (HAM-D score) from week 1 to week 6 was used as outcome in a linear regression model. The model was adjusted for technical variance (the first 5 principal components of the control probes), HAM-D score at admission, age, sex, and DNA methylation at cg05575921 within the aryl hydrocarbon receptor (AHR) repressor gene (*AHRR*) as an established proxy for smoking status (cf. Zeilinger et al., 2013). The threshold for statistical significance was set at *P* < 5.77E-8, corresponding to a Bonferroni correction for the 865 859 evaluated CpG sites (0.05/865 859). Associations at a significance level of *P *< 1E-5 were considered suggestive (Lander and Kruglyak, 1995). The genomic inflation factor lambda (Devlin and Roeder, 1999) was calculated, and QQ-plots were visually inspected (see supplementary Figures 1 and 2). Lambda was 1.06 for the whole sample and 0.97 for the SSRI/SNRI subsample. One of the suggestive CpGs (cg04099436) was flagged for cross-reactivity (cf. Lehne et al., 2015; Pidsley et al., 2016) and thus omitted from the results. None of the CpGs overlapped with single nucleotide polymorphisms (distance to single nucleotide polymorphism ≤5 base pairs and European ancestry–based minor allele frequency ≥0.01).

## RESULTS

**Figure 1.**
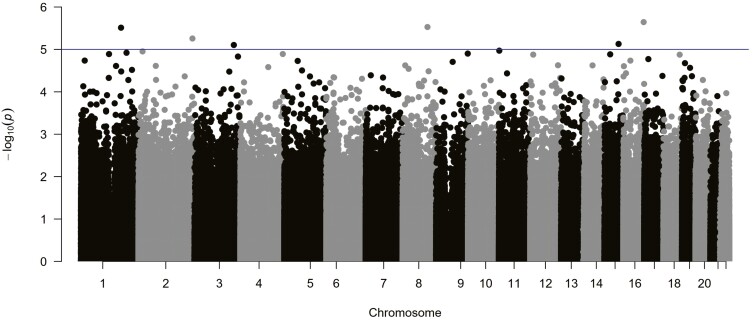
Manhattan plot of the adjusted^a^ epigenome-wide association study (EWAS) in patients with MDD (n = 230) predicting naturalistic antidepressant treatment response after 6 weeks. Horizontal (blue) line: threshold for suggestive CpG sites (*P* < 1E-5). ^a^Adjusted for HAM-D score at admission, age, sex and DNA methylation at cg05575921 within the aryl hydrocarbon receptor (AHR) repressor gene (*AHRR*) as an established proxy for smoking status (cf. Zeilinger et al., 2013).

In the overall sample of MDD patients, no genome-wide significant hits were observed. However, suggestive epigenome-wide evidence was discerned for altered DNA methylation at 6 CpG sites to predict response to a naturalistic antidepressant treatment. These CpGs map to genes affiliated with the long noncoding RNA (lncRNA) class (*LOC102724467*, *LOC100506023*) or coding for the R-Spondin 2 (*RSPO2*), S-Antigen Visual Arrestin (*SAG*), Interleukin 16 (*IL16*), and Protein Kinase C Iota (*PRKCI*) genes (for details, see Table 1 and Figure 1).

**Table 1.** Epigenome-wide DNA methylation predicting antidepressant treatment response in the overall sample of naturalistically treated MDD patients.

CpG	Coefficient (SE)^a^	*T*-value	*P*-value^a^	Nearest gene annotation
cg26573923	-536.57 (110.39)	-4.86	2.27E-06	*LOC102724467*
cg09566189	-89.16 (18.57)	-4.80	2.96E-06	*RSPO2*
cg11091478	-197.87 (41.28)	-4.79	3.07E-06	*LOC100506023 (lnc-TNFSF4-3, PRDX6)*
cg16513582	-217.48 (46.65)	-4.66	5.53E-06	*SAG*
cg26528791	-249.57 (54.32)	-4.59	7.42E-06	*IL16*
cg08510201	359.7 (78.52)	4.58	7.86E-06	*PRKCI*

Response to antidepressant treatment from week 1 to week 6. Results yielding suggestive significance (p<1E-5) are shown. *N* = 230.

Abbreviation: MDD, major depressive disorder.

^a^Analyses are adjusted for HAM-D score at admission, age, sex, and DNA methylation at cg05575921 within the aryl hydrocarbon receptor (AHR) repressor gene (*AHRR*) as an established proxy for smoking status (cf. Zeilinger et al., 2013); a positive coefficient indicates higher methylation to predict increased treatment response.

**Figure 2.**
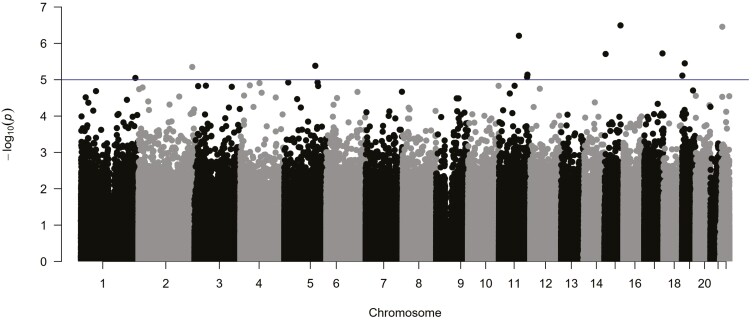
Manhattan plot of the adjusted^a^ epigenome-wide association study (EWAS) in patients with MDD (n = 107) predicting response to SSRI/SNRI treatment after 6 weeks. Horizontal (blue) line: threshold for suggestive CpG sites (*P* < 1E-5). ^a^Adjusted for HAM-D score at admission, age, sex and DNA methylation at cg05575921 within the aryl hydrocarbon receptor (AHR) repressor gene (*AHRR*) as an established proxy for smoking status (cf. Zeilinger et al., 2013).

In patients primarily and continuously treated with SSRIs or SNRIs, differential DNA methylation at 11 CpGs was suggestively associated with treatment response, mapping for instance to the Tissue Metalloproteinase 2 (*TIMP2*), Voltage Dependent Anion Channel 1 (*VDAC1*), or Sortilin Related Receptor (*SORL1*) genes (for details, see Table 2 and Figure 2).

**Table 2.** Epigenome-wide DNA methylation predicting antidepressant treatment response in the subgroup of MDD patients treated with SSRIs/SNRIs.

CpG	Coefficient (SE)^a^	*T*-value	*P*-value^a^	Nearest gene annotation
cg26967050	221.84 (40.17)	5.52	3.2E-07	*MIR9-3HG*
cg03308985	569.22 (103.45)	5.50	3.49E-07	*DGCR6L*
cg12667152	-196.79 (36.67)	-5.37	6.19E-07	*EED*
cg15010903	-224.07 (43.96)	-5.10	1.89E-06	*TIMP2*
cg03608000	686.49 (138.89)	4.94	3.55E-06	*ZNF69*
cg06290379	322.99 (65.86)	4.90	4.14E-06	*VDAC1*
cg11946719	45.22 (9.26)	4.89	4.46E-06	*DIS3L2*
cg11514604	-273.19 (57.35)	-4.76	7.26E-06	*LOC341056*
cg24883899	-462.63 (97.45)	-4.75	7.72E-06	*APC2*
cg25418363	-266.61 (56.44)	-4.72	8.48E-06	*SORL1*
cg00265415	583.49 (123.87)	4.71	8.94E-06	*B3GALNT2*

Response to antidepressant treatment from week 1 to week 6. Results yielding suggestive significance (p<1E-5) are shown. *N* = 107.

Abbreviations: MDD, major depressive disorder; SNRI, serotonin and norepinephrine re-uptake inhibitor; SSRI, selective serotonin re-uptake inhibitor.

^a^Analyses are adjusted for HAM-D score at admission, age, sex, and DNA methylation at cg05575921 within the aryl hydrocarbon receptor (AHR) repressor gene (*AHRR*) as an established proxy for smoking status(cf. Zeilinger et al., 2013); a positive coefficient indicates higher methylation to predict increased treatment response.

## DISCUSSION

While in the present hypothesis-free epigenome-wide association study in MDD patients no epigenome-wide significance was discerned for altered DNA methylation to predict antidepressant treatment response, suggestive evidence was obtained for novel epigenetic candidates possibly to be involved in the prediction of response to antidepressant treatment in a naturalistic in-patient setting. These CpG hits map to genes in systems that have in part been previously implied in depressive or other mental disorder phenotypes: R-Spondin 2 has been implied in the development of midbrain dopaminergic neurons (Gyllborg et al., 2018) and has been linked to fear extinction processes in the amygdala (McCullough et al., 2016). The lncRNA class gene *LOC100506023* originally associated by proximity with CpG cg11091478 was subsequently annotated as *lnc-TNFSF4-3* (Volders et al., 2019). This TNF Superfamily Member 4 (*TNFSF4*) gene has been observed to be downregulated in MDD patients in a genome-wide gene expression analysis (Woo et al., 2018). However, according to the most recent version of GENCODE (Frankish et al., 2021), this CpG maps inside a cis-acting antisense RNA of the adjacent Peroxiredoxin 6 (*PRDX6*) gene. PRDX6 has been linked to depressive (Gu et al., 2022) and anxiety-like (Pairojana et al., 2022) behavior in mice as well as to paroxetine (McHugh et al., 2010), escitalopram, and sertraline (Bisgaard et al., 2012) administration in rats. The pro-inflammatory cytokine interleukin 16 has been shown to reduce neuronal excitability and synaptic activity (Hridi et al., 2019). Additionally, interleukin 16 protein levels have been reported to be altered in a molecular profiling study on serum samples of MDD patients (Stelzhammer et al., 2014) and to be associated with symptom severity in schizophrenia (Schwarz et al., 2012). The Protein Kinase C Iota has been implied in immunosuppression (Sarkar et al., 2017), and genetic variation in the *PRKCI* gene has been found to be associated with bipolar disorder (Iwamoto et al., 2011) and autism (Shen et al., 2011). While the s-antigen visual arrestin is primarily involved in ophthalmological disorders, it has been shown to also be expressed in nonphotosensitive cells controlled by the beta adrenergic G-protein–mediated adenylate cyclase system (Faure and Mirshahi, 1990) and might be linked to T-cell activation and cytokine response in healthy probands and thus potentially also to immune-mediated processes relevant to depression (see Morgan et al., 2002). The lncRNA gene *LOC102724467* has not previously been linked to mental disorder phenotypes.

In the subsample of patients primarily and continuously treated with SSRIs/SNRIs, suggestive evidence was revealed for further epigenetic candidates to possibly predict treatment response. For instance, the DiGeorge critical region 6 and its duplication (*DGCR6L*) have been shown to interact with the gamma-aminobutyric acid GABA-B1 receptor subunit (Zunner et al., 2010) and to be related to anxiety in children with chromosome 22q11.2 deletion syndrome (Das Chakraborty et al., 2012) as well as to schizophrenia (Liu et al., 2002). The embryonic ectoderm development gene (*EED*) codes for a core component of the polycomb-repressive complex 2, which has been linked to neurodevelopmental abnormalities, intellectual disability, and neurodegeneration, and microglial EED has been suggested to play a pivotal role in normal synaptic and cognitive functions (Wang et al., 2022). A genetic polymorphism in the *TIMP2* gene coding for the tissue metalloproteinase 2 modulating inflammation was found to be associated with depression (Bobińska et al., 2016b), and increased TIMP-2 mRNA levels have been observed in patients with depression (Bobińska et al., 2016a). Altered methylation of the Zinc Finger Protein 69 (*ZNF69*) gene has been suggested to play a role in neurogenesis and brain development in Down syndrome (Laan et al., 2020). The Voltage Dependent Anion Channel 1 (*VDAC1*) has been shown to be downregulated in rats resilient to chronic social isolation stress (Perić et al., 2021) and in response to learned helplessness (Li et al., 2016), both constituting animal models of depression. A duplication of the APC Regulator of WNT Signaling Pathway 2 (*APC2*) gene has been found to be protective against developing Alzheimer disease with psychotic symptoms (Zheng et al., 2015). The Sortilin Related Receptor (*SORL1*) also known as SorLA-1 has been suggested to be implicated in major depression, and its treatment as SorLA level increased significantly in a subgroup of patients treated with nortriptyline, but interestingly, and contrary to the present results, not with escitalopram (Buttenschøn et al., 2018). The MIR9-3 Host Gene (*MIR9-3HG*), the lncRNA class-associated *LOC341056* gene, and the Beta-1,3-N-Acetylgalactosaminyltransferase 2 (*B3GALNT2*) have not been reported to be related to a mental disorder phenotype before. While these presently identified hits add to the available literature on potential biomarkers of MDD and its treatment response, the functional role of altered DNA methylation at the above-mentioned gene loci in predicting antidepressant treatment response remains to be elucidated in future studies.

The presently identified hits did not overlap with CpGs associated with antidepressant treatment response in previous EWAS (cf. Takeuchi et al., 2017; Ju et al., 2019; Martinez-Pinteno et al., 2021; Engelmann et al., 2022). However, previous sample sizes were very small and differed both in sample composition (e.g., age, ethnicity) and statistical approaches, thus limiting comparability.

Additionally, results were compared with those from genome-wide association meta-analyses (Fabbri et al., 2019; Li et al., 2020), which did not yield any overlap with the genes identified in the present study.

The presently reported results have to be considered in light of some limitations. Epigenome-wide DNA methylation was determined in peripheral blood samples, thus precluding direct conclusions regarding central processes. The present results therefore need further investigation regarding their validity as potential surrogates for central processes, for instance, utilizing postmortem human brain samples, animal models, or in silico analysis tools such as BECON (Edgar et al., 2017), IMAGE-CpG (Braun et al., 2019), or the Blood Brain DNA Methylation Comparison Tool (Hannon et al., 2015), which, however, do not include all CpGs covered by the Infinium MethylationEPIC BeadChip as presently applied or cover all brain regions relevant to MDD or antidepressant treatment response.

While all patients included in the present study received serotonergic antidepressants as their primary medication, given the naturalistic study design, varying drug classes of differing dosage were used, and dosages were not held constant over the course of treatment. Additionally, drug initiation or discontinuation during the observation period was allowed, and co-medication with other pharmacological agents (e.g., mood stabilizers, antipsychotics) was permitted, with the exception of MAO inhibitors or valproic acid, both of which are known to affect DNA methylation (for review see Boks et al., 2012). Still, co-medication cannot be excluded as a potential confounding source on the present results. Along those lines, drug effects themselves may represent a confounding variable. However, cross-referencing of the presently identified hits suggestively predicting antidepressant treatment response with the 10 CpGs reported to be associated with antidepressant use in a recent EWAS (Barbu et al., 2022) did not reveal any overlap. The presently identified suggestive associations may also be specific to pharmacological treatment as there was no overlap with findings from a methylome study in electroconvulsive therapy (Sirignano et al., 2021). However, replication and preferably directly comparable future studies are needed to address the common and diverging epigenetic signatures associated with different treatment options in MDD. While smoking status has been reported to affect global DNA methylation levels (Gao et al., 2015), information on smoking was not available for the present sample. However, correction for methylation at cg05575921 within the aryl hydrocarbon receptor (AHR) repressor gene (*AHRR*) as an established proxy for smoking status (cf. Zeilinger et al., 2013) was applied in the presently described treatment prediction analyses to account for the potentially confounding effect of nicotine smoking. While the present size constitutes the largest sample investigated in this regard, the sample size is overall still moderate and may be underpowered to detect small effect sizes, warranting validation and replication in even larger samples. Future studies may also want to take into account clinical characteristics such as chronicity or previous treatment attempts in adequately powered samples. Finally, the suggestive hits identified here would ideally be investigated in conjunction with genetic variants using an integrated EWAS-GWAS approach to fully understand their combined effects in appropriately powered samples.

Taken together, the present results provide preliminary suggestive evidence for novel epigenetic candidates to possibly be associated with antidepressant treatment response in MDD in a naturalistic in-patient treatment setting. Given the high rates of treatment nonresponse and treatment resistance in MDD and associated burden of disease, the identification of valid and objective biomarkers to predict treatment response constitute a cornerstone of current clinical research toward improving clinical decision-making and providing more personalized treatments for MDD in an effort to increase treatment response rates. While epigenetic research into treatment response is still in its infancy and not yet ready for translation into clinical practice, it is hoped to inform prognosis and treatment decisions in the future within a personalized medicine framework.

## Supplementary Material

pyae045_suppl_Supplementary_Figures

## Data Availability

Data can be made available upon reasonable request from the corresponding author.

## Interest Statement

K.D. is a member of the Neurotorium Editorial Board, Lundbeck Foundation, was a member of the Janssen Pharmaceuticals, Inc. Steering Committee Neurosciences, and received speakers’ honoraria from Janssen Pharmaceuticals, Inc. within the past 3 years. B.T.B. is a member of Advisory Boards—Lundbeck, Janssen-Cilag; Consultant—National Health and Medical Research Council, Australia; Grant/Research Support—AstraZeneca, Fay Fuller Foundation, James & Diana Ramsay Foundation, National Health and Medical Research Council, Australia; German Research Council (DFG), European Union, EraPerMEd, Wellcome Trust; B.T.B. has received honoraria—AstraZeneca, Bristol-Myers Squibb, Lundbeck, Pfizer, Servier Laboratories, Wyeth Pharmaceuticals, Takeda, Janssen, LivaNova PLC, Boehringer-Ingelheim. V.A. has received compensations for his contributions as member of advisory boards and for presentations for the following companies: Astra-Zeneca, Eli Lilly, Janssen-Cilag, Lundbeck, Otsuka, Sanofi, Servier, and Trommsdorff, and has received funding from Horizon 2020 EU 3.1.1 (Project MOODSTRATIFICATION). These cooperations have no relevance to the work that is covered in the manuscript. P.Z. has received speaker fees or honoraria for advisory board participation from Janssen Pharmaceuticals, Schwabe, Servier, and Neuraxpharm. All other authors have no conflicts of interest to declare.
