# Supplementary material for: Epigenome-Wide DNA Methylation in Unipolar Depression: Predictive Biomarker of Antidepressant Treatment Response?
Source: Int J Neuropsychopharmacol. 2024 Oct 5;27(11):pyae045. doi: 10.1093/ijnp/pyae045 (PMC11558245; doi:10.1093/ijnp/pyae045)
Supplement: pyae045_suppl_Supplementary_Figures [file pyae045_suppl_supplementary_figures.pdf]

Supplementary Figure S1. QQ-Plot for the adjusted[a] epigenome-wide association study (EWAS) in patients with MDD (N=230) predicting naturalistic antidepressant treatment response after six weeks

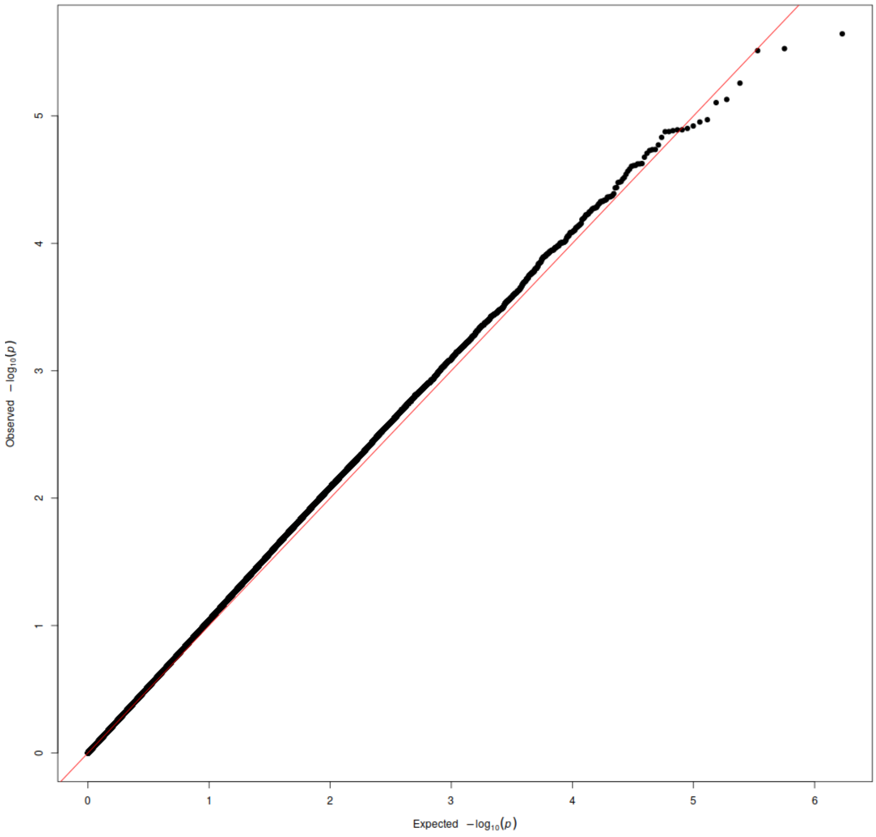

Legend to Supplementary Figure S1: Quantile-Quantile (QQ) plot visualizing the deviation of the observed -log10-transformed p-values from the theoretical null distribution (inflation). The inflation factor corresponding to the plot is lambda=1.0599. [a] adjusted for HAM-D score at admission, age, sex and DNA methylation at cg05575921 within the aryl hydrocarbon receptor (AHR) repressor gene (*AHRR*) as an established proxy for smoking status (cf. Zeilinger et al., 2013).

Supplementary Figure S2. QQ-Plot for the adjusted[a] epigenome-wide association study (EWAS) in patients with MDD (N=107) predicting SSRI/SNRI treatment response after six weeks

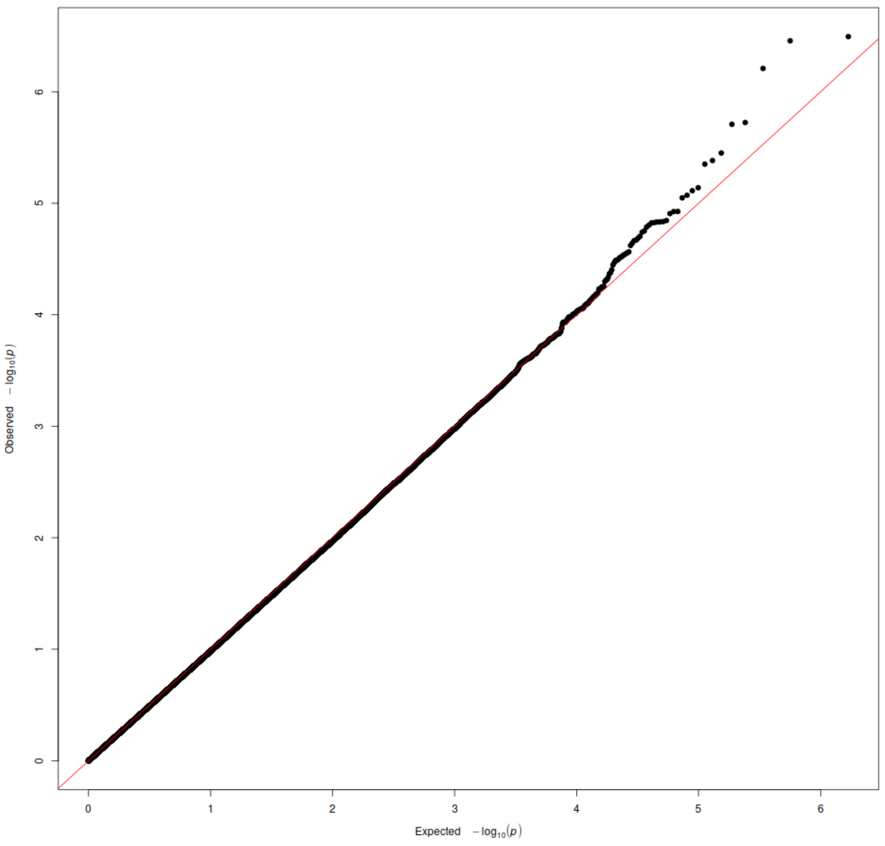

Legend to Supplementary Figure S2: Quantile-Quantile (QQ) plot visualizing the deviation of the observed -log10-transformed p-values from the theoretical null distribution (inflation). The inflation factor corresponding to the plot is lambda=0.9689. [a] adjusted for HAM-D score at admission, age, sex and DNA methylation at cg05575921 within the aryl hydrocarbon receptor (AHR) repressor gene (*AHRR*) as an established proxy for smoking status (cf. Zeilinger et al., 2013).
